# Sourcing of human peripheral blood-derived myeloid angiogenic cells under xeno-free conditions for the treatment of critical limb ischemia

**DOI:** 10.1186/s13287-022-03095-5

**Published:** 2022-08-13

**Authors:** Christy Wing Tung Wong, Apurva Sawhney, Yalan Wu, Yi Wah Mak, Xiao Yu Tian, Hon Fai Chan, Anna Blocki

**Affiliations:** 1 Institute for Tissue Engineering and Regenerative Medicine, The Chinese University of Hong Kong, Shatin, Hong Kong SAR China; 2 School of Biomedical Sciences, The Chinese University of Hong Kong, Shatin, Hong Kong SAR China; 3 Department of Orthopaedics & Traumatology, Faculty of Medicine, The Chinese University of Hong Kong, Shatin, Hong Kong SAR China

**Keywords:** Myeloid angiogenic cells, Blood-derived angiogenic cells, Mesenchymal stem cells, Therapeutic angiogenesis, Critical limb ischemia, Xeno/serum free, Revascularization

## Abstract

**Background:**

Critical limb ischemia (CLI) is the most severe form of peripheral artery disease and exhibits a high risk of lower extremity amputations. As even the most promising experimental approaches based on mesenchymal stem cells (MSCs) demonstrated only moderate therapeutic effects, we hypothesized that other cell types with intrinsic roles in angiogenesis may exhibit a stronger therapeutic potential. We have previously established a protocol to source human peripheral blood-derived angiogenic cells (BDACs). These cells promoted revascularization and took perivascular location at sites of angiogenesis, thus resembling hematopoietic pericytes, which were only described in vivo so far. We thus hypothesized that BDACs might have a superior ability to promote revascularization and rescue the affected limb in CLI.

**Methods:**

As standard BDAC sourcing techniques involve the use of animal-derived serum, we sought to establish a xeno- and/or serum-free protocol. Next, BDACs or MSCs were injected intramuscularly following the ligation of the iliac artery in a murine model. Their ability to enhance revascularization, impair necrosis and modulate inflammatory processes in the affected limb was investigated. Lastly, the secretomes of both cell types were compared to find potential indications for the observed differences in angiogenic potential.

**Results:**

From the various commercial media tested, one xeno-free medium enabled the derivation of cells that resembled functional BDACs in comparable numbers. When applied to a murine model of CLI, both cell types enhanced limb reperfusion and reduced necrosis, with BDACs being twice as effective as MSCs. This was also reflected in histological evaluation, where BDAC-treated animals exhibited the least muscle tissue degeneration. The BDAC secretome was enriched in a larger number of proteins with pro-angiogenic and anti-inflammatory properties, suggesting that the combination of those factors may be responsible for the superior therapeutic effect.

**Conclusions:**

Functional BDACs can be sourced under xeno-free conditions paving the way for their safe clinical application. Since BDACs are derived from an easily accessible and renewable tissue, can be sourced in clinically relevant numbers and time frame and exceeded traditional MSCs in their therapeutic potential, they may represent an advantageous cell type for the treatment of CLI and other ischemic diseases.

## Background

Peripheral artery disease (PAD) arises from dysfunctional macro- and microcirculation in lower extremities, which leads to impaired blood supply to peripheral tissues and results in hypoxia, inflammation, vascular dysfunction and muscle degeneration. PAD prevalence and incidence are both sharply age-related, rising to > 10% among people in their 60s and 70s. The most severe form of PAD is critical limb ischemia (CLI) and occurs in 11% of PAD patients [1, 2], who then experience rest pain, non-healing ulcers, gangrene and are at high risk of lower extremity amputations [3]. Hence, CLI is a major cause of disability and a major socioeconomic burden globally [3].

Conventional therapeutic strategies include establishment of reperfusion by bypass surgery, angioplasty, stenting, as well as pharmacological approaches [4, 5]. Unfortunately, interventions based on reperfusion do not restore blood flow entirely and do not promote the repair of the macro- and microvasculature [6]. Furthermore, approximately 50% of patients are not eligible for reperfusion therapy, resulting in poor outcomes [7, 8].

Various experimental approaches attempted to remedy this and have covered biologics- or gene-based, as well as cell-based approaches, as independent protocols or incorporated into various types of delivery systems [7, 9, 10]. During protein or related gene therapy, pro-angiogenic factors are introduced into the affected tissue to stimulate local angiogenesis [3, 9]. However, resulting supra-physiological doses of these factors are associated with various adverse effects. Furthermore, strategies based on the delivery of a few growth factors do not sufficiently capture the complexity of signals and temporal coordination required to direct such intricate biological processes [7, 9].

The delivery of cells promises a more holistic approach, whereby cells may locally secrete a wide range of pro-regenerative factors at physiologically relevant levels [11]. Here the clinically most investigated cell type is mesenchymal stem cells (MSCs) isolated from bone marrow followed by those derived from adipose tissue [11–13]. Cell-based therapy has been shown to be safe, while resulting in a spectrum of outcomes ranging from no therapeutic effect to being reasonably effective in slowing the progression of the disease and improving the quality of life of patients [11].

Nonetheless, MSC-based therapies face many challenges, as the hostile microenvironment in ischemic tissue also compromises cellular survival upon implantation. Furthermore, autologous MSC-based therapies require a long preparation time, which can be prohibitively long to meet a therapeutic window [14, 15].

MSCs have an extensive historical background in being explored for the treatment of various pathologies [16]. However, MSCs may not prove to be the solution for the treatment of all pathologies, and other cell types with intrinsic roles in angiogenesis are worth exploring in therapeutic approaches to treat ischemic diseases.

Angiogenesis is a highly synchronized process involving several cell types. During the initial stages, the vascular basement membrane is degraded and mesenchymal pericytes, which are in essence MSCs [17, 18], detach from the vessel wall. The hypoxic tissue produces a gradient of pro-angiogenic factors, toward which the activated endothelial cells proliferate and migrate. Following the gradient, the endothelial cells re-arrange to form new tubular structures that eventually become re-enveloped with mesenchymal pericytes and basement membrane [19]. There are various additional supportive cell types involved in the angiogenic and arteriogenic processes including myeloid cells [7]. In particular, hematopoietic pericytes have gained some attention recently [20, 21]. They were identified proximally to sprouting endothelial cells during angiogenesis, based on their perivascular location and the expression of pericytic markers, as well as hematopoietic and monocytic markers such as PDGFRβ, NG2, as well as CD45, CD11b or F4/80, respectively [22–27]. Hematopoietic pericytes have been concluded to guide angiogenesis and anastomosis, denoting their major role in guiding and driving angiogenesis [23]. In contrast, mesenchymal pericytes, thus MSCs, are responsible for microvessel maturation, stabilization and quiescence [28]. The absence of mesenchymal pericytes during angiogenesis had no effect on microvessel density [23, 29], suggesting them to not be involved in the initial stages of angiogenesis. Hence, hematopoietic pericytes may indeed be the more appropriate cell type to promote revascularization.

It is therefore not surprising that various myeloid cell types have been investigated for their utilization in therapeutic angiogenesis. Those can be summarized as myeloid angiogenic cells (MACs) and have been published under various nomenclature such as fibrocytes, early endothelial progenitor, endothelial-like or endothelial colony-forming cells (early EPCs, ELCs or ECFCs). Importantly, MACs have to be clearly distinguished from true EPCs first isolated by Asahara et al. [30], also known as late EPCs or outgrowth endothelial cells (OECs) [31], as the latter are true building blocks of the inner lining of the vasculature.

We have previously established a protocol to source human peripheral blood-derived angiogenic cells (BDACs) with monocyte and pericyte characteristics in vitro [24]. BDACs have demonstrated a pro-angiogenic potential in vitro and in vivo and take perivascular location at sites of angiogenesis, thus resembling hematopoietic pericytes, only described in vivo thus far [23]. BDACs share markers and secreted factors with other MACs, but also exhibit clear differences such as lack of VE-Cadherin and collagen type I as common markers for early EPCs and fibrocytes, respectively [24]. Furthermore, their intramuscular injection around the ligation site in a murine model of CLI accelerated revascularization, thereby rescuing the affected limb from extensive necrosis. Since BDACs are derived from an easily accessible and renewable tissue, as well as can be sourced in clinically relevant numbers and time frame, they represent a promising cell type to be utilized in therapeutic angiogenesis approaches [24].

Unlike MSCs, MACs such as BDACs are a relatively new cell type explored for cell-based therapies and their sourcing and in vitro culture are not yet established under Good Manufacturing Practices (GMP), but rather based on standard culture conditions with fetal bovine serum (FBS) supplemented culture medium. Indeed, the serum provides a broad spectrum of attachment proteins, growth factors, macromolecules and many more [32]. However, a major drawback of the use of serum is the wide range of possible contaminants which can give rise to animal-derived diseases and trigger immunogenic responses [33, 34]. Serum also exhibits large batch-to-batch variabilities, which lead to differences in cell growth and quality [35]. Hence, our team has sought to establish a xeno-free (XF) and/or serum-free (SF) BDAC sourcing protocol using a commercially available medium that would allow to maintain cell numbers as well as functionality. These BDACs were then investigated in a preclinical CLI model and compared to the current state-of-the-art cell-based therapy based on bone marrow-derived MSCs.

## Materials and methods

### Isolation of peripheral blood mononucleated cells (PBMCs)

All experiments were approved by the Joint Chinese University of Hong Kong-New Territories East Cluster Clinical Research Ethics Committee, Hong Kong (Ref No: 2019.353). Upon receiving consent, 20–40 ml of peripheral blood were collected from healthy donors. PBMCs were isolated from these healthy human peripheral blood samples using a Lympho/PBMC spin medium (cat. #60-00092-12; pluriSelect Life Science UG, Leipzig, Germany) accordingly to manufacturer’s instructions.

### Sourcing of BDACs

PBMCs were seeded at 2 million cells/ml and 0.5 million cells/cm^2^ in various media as listed in Table 1, which were further supplemented with a Ficoll cocktail (Fc400 25 mg/ml; cat. #17-0300-50 and Fc70 37.5 mg/ml; cat. #17-0310-10, Sigma-Aldrich, St. Louis, MO, USA), on fibronectin (cat. #341635**;** Sigma-Aldrich, St. Louis, MO, USA)-coated dishes at 2 µg/cm^2^ accordingly to a previously published protocol [24]. After 24 h, media and non-adherent cells were removed, and respective media without Ficoll macromolecules were added. Adherent cells were cultured further for 4–7 days.

**Table 1:** Choice of culture media investigated for BDAC sourcing

Culture media	Short form	Xeno free/serum free
Dulbecco's modified Eagle medium with 10% fetal bovine serum (FBS) and Penicillin–Streptomycin (P/S), Standard media (established protocol) Gibco (DMEM, cat.# 31600092; FBS, cat. #16000044; P/S, cat. #15140-122; Life Technologies, Grand Island, NY, USA)	DMEM/FBS	–
CnT-Prime MSC Proliferation Medium, Xeno-Free CELLnTEC Advanced Cell Systems AG (cat. #CnT-PR-MSC-XF; Stauffacherstrasse, Bern, Switzerland)	CnT	Xeno free
Human Stem X Vivo serum-free MSC expansion media R&D systems (cat. #CCM014; Minneapolis, USA)	StemXVivo	Serum free
MSC NutriStem XF medium (supplemented with PLTGold Human Platelet Lysate, research grade) Sartorius AG (cat. #05-200-1A, #PLTGOLD100R; Goettingen, Germany)	NutriStem	Xeno free
TheraPEAK Mesenchymal Stem Cell Growth Medium Lonza (cat. #BEBP18-936; Basel, Switzerland)	TheraPEAK	Chemically defined
Mesenchymal Stem Cell growth medium DXF PromoCell (cat. #C-28019; Heidelberg, Germany)	DXF	Xeno free, serum free

### Immunocytochemistry

BDACs were fixed with 100% ice-cold methanol and then blocked in 3% bovine serum albumin (BSA) in PBS for 1 h. Next, samples were incubated with respective primary antibodies obtained from Abcam (Hong Kong, HK SAR); recombinant Anti-NG2 (NG2, 1:250; cat. #ab255811, Abcam) and recombinant Anti-PDGFR-β antibody (PDGFR-β, 1:100; cat. #ab32570, Abcam) in 1% BSA/PBS overnight at 4 °C. After washing all samples three times using PBS, secondary antibodies Alexa Fluor 488 (1:500; cat. #ab150077, Abcam) and 4’, 6-diamidino-2-phenylindole (DAPI 1:1,000, cat. #564907, BD Pharmingen, San Diego, CA, USA) in 1% BSA/PBS were added and incubated for 90 min at room temperature. Samples incubated without primary antibodies acted as conjugate controls. Fluorescent staining was visualized using an Olympus IX83 inverted fluorescence microscope (Olympus, Tokyo, Japan) equipped with cellSens Dimension image acquisition software (Olympus, Tokyo, Japan). All images were analyzed by ImageJ software (https://imagej.nih.gov/ij/).

### Flow cytometry

Adherent cells were harvested by trypsinization. Cells were suspended in 1% BSA/PBS buffer at a concentration of 10^5^ cells/100 μl. Cell suspensions were incubated with BV711 Mouse Anti-Human CD45 (1:20; cat. #5643257, BD Biosciences, Franklin Lakes, NJ, USA) and APC Mouse Anti-Human CD11b (1:5; cat. #550019, BD Biosciences, Franklin Lakes, NJ, USA) for 30 min at 4 °C in the dark. Cells were washed 2 times with 1%PBS/BSA, resuspended in 1 ml 1%PBS/BSA and analyzed by BD FACS Aria II Cell Sorter (BD Life Sciences, San Jose, CA, 95131, USA). Unstained cells were used as negative controls.

### Spheroid sprouting assay

This functional assay was adapted from previously published protocols [9, 24, 36, 37]. 6 × 10^5^ green fluorescent protein labeled human umbilical vein endothelial cells (HUVECs; cat. #P20201; Innoprot, Bizkaia, Spain) were seeded in fully supplemented EGM-2 medium (cat. #CC-3162; Lonza, Basel, Switzerland) into a well in an AggreWell™ 400 plate overnight, allowing spheroids with an average number of 500 cells per spheroid to form. On the next day, 350 spheroids were resuspended in 0.3 ml EGM-2. This suspension, optionally supplemented with 3.5 × 10^4^ BDACs/cm^2^, where indicated, were mixed with neutralized 0.6 ml of 2 mg/ml collagen I TeloCol®-6 hydrogel (cat. #5225; Advanced BioMatrix, Carlsbad, USA) one-to-one and cast in a 12-well plate well. The collagen hydrogels were allowed to polymerize for 2 h at 37 ºC, upon which they were layered with 0.7 ml EGM-2. Spheroids were allowed to sprout for 1–2 days, after which pictures were taken with an epifluorescence microscope (Olympus IX83, Olympus, Tokyo, Japan) equipped with cellSens Dimension image acquisition software (Olympus, Tokyo, Japan). The cumulative sprout length per spheroid was measured, processed and quantified by ImageJ v1.52i software (https://imagej.nih.gov/ij/).

### Mesenchymal stem cell culture

Human bone marrow mesenchymal stem cells (Human bmMSCs; cat. #SCC034; Millipore Temecula, CA, USA) at passage 5 were thawed and cultured in low glucose Dulbecco Modified Eagle Medium (DMEM, cat. #31600092; Gibco, Life Technologies, Grand Island, NY, USA) supplemented with 10% fetal bovine serum (FBS, cat. #16000044; Gibco, Life Technologies, Grand Island, NY, USA) and 1% Penicillin–Streptomycin (P/S, cat. #15140-122; Gibco, Life Technologies, Grand Island, NY, USA). Subculture included trypsinization with TrypLETM Express (cat. #12605-010; Gibco, Life Technologies, Grand Island, NY, USA) and centrifugation at 25℃ for 5 min with 300 relative centrifugal force (RCF).

### Proteome profiler cytokine array

Blood-derived adherent cells and MSCs were incubated for 24 h in DMEM supplemented with 0.5% FBS to produce a conditioned medium at a concentration of 115,000 cells/cm^2^. A proteome profiler human XL cytokine array kit (cat. #ARY022B; R&D Systems, Minneapolis, USA) was used accordingly to the manufacturer’s instructions to identify a wide range of cytokines secreted by both cell types. All antibodies and reagents were provided within the kit. The samples were diluted with blocking buffer and incubated overnight with a pre-blocked cytokine array membrane. The membrane was washed and then incubated with biotinylated detection antibodies. Streptavidin-HRP and chemiluminescent detection reagents were used. Signals corresponding to different cytokines were captured by the Bio-Rad ChemiDoc Imaging System (Bio-Rad Laboratories, Inc., CA, USA), and the profile of mean spot pixel density was measured by creating a region on interest (ROI) for each spot using ImageJ v1.52i software (https://imagej.nih.gov/ij/). Each spot density was normalized to the average mean value of the positive controls. The average value of BDACs was then divided by the average value of MSCs of each cytokine and shown as fold-change of BDACs to MSCs in a heatmap using GraphPad Prism v8.0 (GraphPad Software, San Diego, CA, USA, www.graphpad.com).

### In vivo murine hindlimb ischemia model

All animal experiments were approved by the Animal Ethics Experimentation Committee at the Chinese University of Hong Kong (Ref. No. 20-261_ITF). Unilateral hindlimb ischemia was induced in balb/c nude mice (male, 8–10 weeks, 18–25 g, *n* = 6 per treatment group). Briefly, the mice were anesthetized by intraperitoneal injection of an anesthetic mixture of Ketamine (10%) and Xylazine (2%) in 0.9% sodium chloride solution, using 0.1 mL per 10 g animal body weight. After a small incision, the external iliac artery was separated from the surrounding tissue and ligated with a 7–0 polypropylene suture (Hangzhou Huawei Medical Instruments Co., Ltd., Hangzhou, China). Animals received 2 injections of 20 µl into the muscle tissue adjacent to the ligation site and the gastrocnemius muscle, respectively. Each animal received a total of 250,000 of MSCs or BDACs. The incision was then closed with a 4–0 polyglycolic acid suture. The mice received subcutaneous injection of Buprenorphine (0.1 mg/kg) twice per day for 3 days post-surgery.

#### Laser speckle imaging and analysis

Limb perfusion of both limbs was imaged using Laser Speckle Contrast Imaging (RFLSI III Laser Speckle Contrast Imaging System, RWD Systems, CA, USA) on days 1, 4, 8 and 15 post-surgery. Animals were anesthetized by intraperitoneal injection of anesthetic mixture of Ketamine (10%), Xylazine (2%) in 0.9% sodium chloride solution, using 0.1 mL per 10 g animal body weight. Lower extremities of mice were imaged across several blood perfusion exposures along with real-time images. Briefly, regions of interest (ROIs) in the lower paw vessels were segmented in both limbs. The mean intensity of the Red pixels was measured by ImageJ software (https://imagej.nih.gov/ij/) to quantify the blood perfusion in the limb areas. The perfusion recovery rate was hence devised as a ratio of the Red pixel intensities between ROIs of ischemic and non-ischemic limbs and finally expressed as a percentage.

#### Ischemic score analysis

A modified ischemic score was also defined to allow for visually assessing and semi-quantifying the paw discoloration by using the real-time limb images taken by the Laser Speckle Contrast Imaging (RFLSI III Laser Speckle Contrast Imaging System, RWD Systems, CA, USA) on days 1, 4, 8 and 15.

#### Histological staining and analysis

Mice gastrocnemius muscle tissues from each treatment group were harvested, fixed with paraformaldehyde and paraffin-embedded for histological staining. Hematoxylin–eosin (H&E) staining allowed to visualize the tissue morphology, presence of neutrophil infiltration (indicator of inflammation) and adipose replacement (muscle degeneration). Masson’s trichrome (M&T) staining allowed for the differentiated visualization of fibrosis by staining the collagen fibers (which are a major component of fibrotic tissue) in blue. Histological staining was performed according to standard protocols [38, 39], and photographs were taken in bright-field microscopy using a Nikon Eclipse Ni-U microscope (Nikon, Tokyo, Japan).

#### Immunohistochemistry

Mice gastrocnemius muscle tissues were embedded into paraffin blocks for microtome sectioning. The samples were cut into 10 µm microsections on slides using the Lateral position Rotary Microtome (Leica RM 2235, Leica Biosystems, Nussloch GmbH, Germany). Subsequently, samples were put into an antigen retriever (PT Module, Thermo, Massachusetts, USA) for antigen retrieval. The antigen retrieved samples were washed in TBS with 0.025% Triton X-100 for 5 min twice. Blocking was achieved using 3% BSA in TBS and 0.3 M of glycine for 2 h at room temperature. After blocking, antibodies used for immunohistochemistry staining (Recombinant Anti-CD11b (CD11b, 1:100; cat. #MA1-80091, Invitrogen, Massachusetts, USA); Recombinant Anti-CD31 (CD31, 1:50, cat. #AF3628, R&D System, Minneapolis, USA); Recombinant Anti-CD206 (CD206, 1:100, cat. #ab64693, Abcam, Hong Kong, HK SAR) and Recombinant Anti-iNOS (iNOS, 1:20, cat. #PA1-036, Thermo Fisher Scientific, Massachusetts, USA) were incubated with the specimens in 1% BSA/TBS overnight at 4 °C. After washing all samples using TBS with 0.025% Triton X-100 for 5 min twice, secondary antibodies Alexa Fluor 488 (1:500; cat. #ab150077, Abcam, Hong Kong, HK SAR), Alexa Fluro 594 (1:500; cat. #ab150160, Abcam, Hong Kong, HK SAR) and Alexa Fluor 488 (1:500; cat. #ab150129, Abcam, Hong Kong, HK SAR) in 1% BSA/TBS were added and incubated for 1 h at room temperature. Samples incubated without primary antibodies acted as conjugate controls. Nuclear counterstaining was performed using 4′,6-diamidino-2-phenylindole (DAPI 1:1,000, cat. #564907, BD Pharmingen, San Diego, CA, USA). Slides were mounted with ProLong gold antifade mountant (cat# P36930, Thermo Fisher Scientific, Massachusetts, USA). Images were captured using Olympus IX83 inverted fluorescence microscope (Olympus, Tokyo, Japan) equipped with cellSens Dimension image acquisition software (Olympus, Tokyo, Japan).

#### Statistical analysis

All in vitro experiments were performed at least three times independently. Values obtained were averaged and displayed as average value ± SD. One-way analysis of variance algorithm was used, and p values below 0.05 were considered statistically significant. The analysis was performed using GraphPad Prism v8.0 (GraphPad Software, San Diego, CA, USA, www.graphpad.com).

## Results

### BDACs can be derived under xeno-free conditions

In order to source BDACs under xeno-/serum-free (XF/SF) conditions, we utilized commercially available media (Table 1), previously optimized for MSC culture, to replace DMEM supplemented with FBS (DMEM/FBS), while all other components of the sourcing protocol remained unchanged. This involved culturing of PBMCs on fibronectin-coated dishes in media supplemented with mixed Ficoll macromolecules (70 kDa and 400 kDa) [10, 24, 40, 41]. After one day, culture media together with non-adherent cells were removed and replaced by fresh media without Ficoll macromolecules. Cells were then matured for another 4–7 days.

**Fig. 1:**
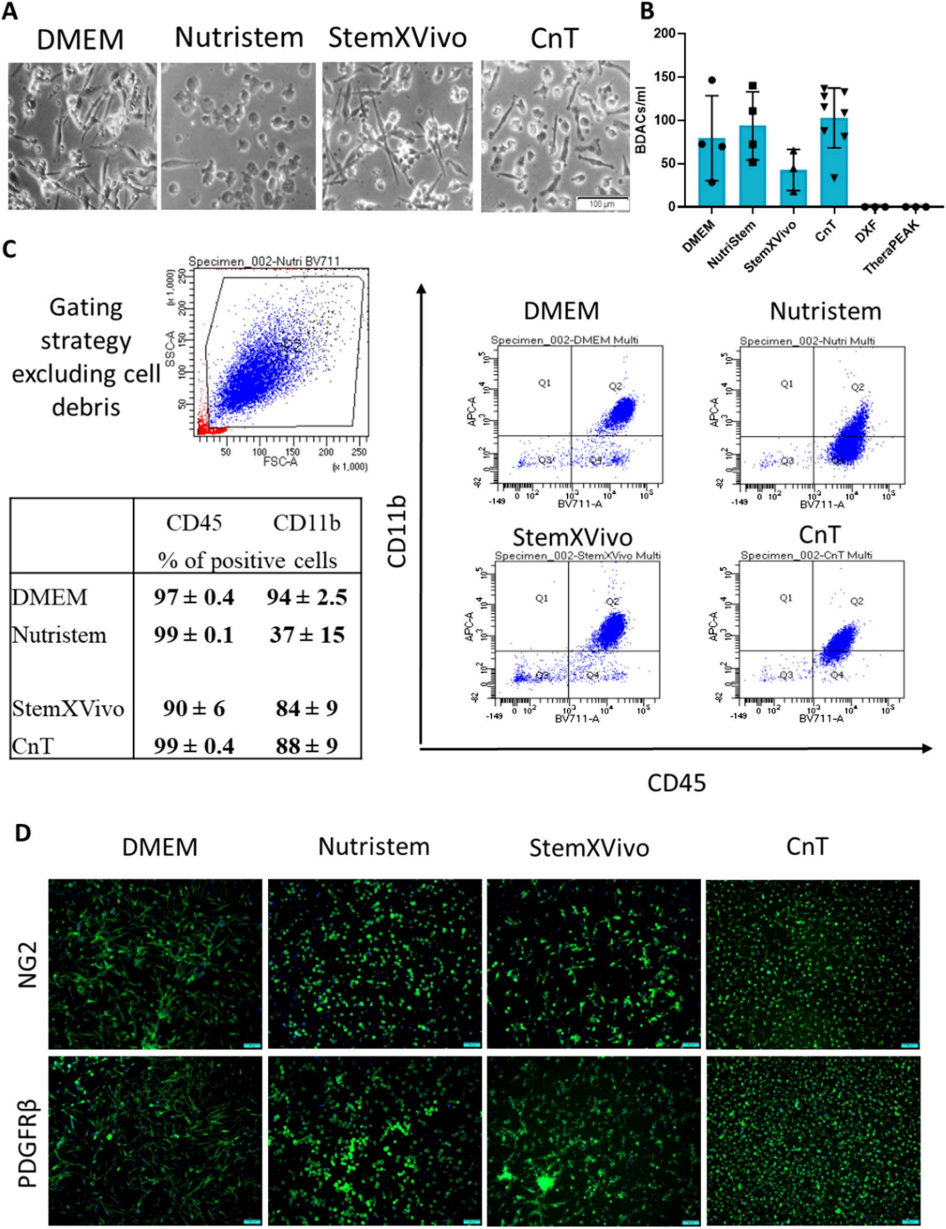
Adherent cells sourced from PBMCs under XF/SF conditions exhibited BDAC characteristics. **A** Phase contrast images of adherent cells sourced from PBMCs under XF/SF conditions after 7 days of culture. **B** Number of adherent cells per initial volume of peripheral blood after 7 days of culture. **C** Flow cytometry analysis for hematopoietic marker CD45 and CD11b expression by adherent cells sourced from PBMC cultured in various media (*n* = 3). **D** Immunocytochemistry for pericyte markers NG2 and PDGFR-β expressed by adherent cells sourced from PBMC cultured in various culture media

As expected, cells isolated under standard culture conditions (DMEM/FBS) exhibited a spindle-shaped morphology (Fig. 1A). A similar morphology was observed for cells cultured in StemXVivo medium (Fig. 1A), although a 50% reduction in the number of resulting adherent cells was observed (Fig. 1B). Despite this considerable decline, differences in cell numbers did not exhibit statistical significance, due to high variance between the blood sample batches. Sourcing cells in CnT medium also resulted in the formation of cells with spindle-shaped morphology, although cells appeared shorter, whereas the majority of cells sourced with NutriStem media exhibited a round cell morphology (Fig. 1A). Densities of adherent cells cultured in NutriStem and CnT were comparable to cells sourced in DMEM/FBS (Fig. 1B). Comparatively, culturing PBMCs with DXF and TheraPEAK media resulted in no adherent cells (Fig. 1B). Hence, the latter two media were excluded from further studies.

BDACs have been previously known to express leukocyte marker CD45, monocyte maker CD11b, as well as pericyte markers PDGFR-β and NG2 [24]. We thus investigated adherent cells cultured in various media for the expression of these markers. Adherent cells cultured in all media expressed comparable and robust levels of CD45 (Fig. 1C). Cells supplemented with DMEM/FBS, StemXVivo and CnT all expressed high levels of CD11b, except for cells sourced in NutriStem which exhibited a more than 50% reduction in CD11b^+^ cells overall (Fig. 1C). All cells exhibited positive staining for pericyte markers NG2 and PDGFR-β, independent of the culture media employed (Fig. 1D).

**Fig. 2:**
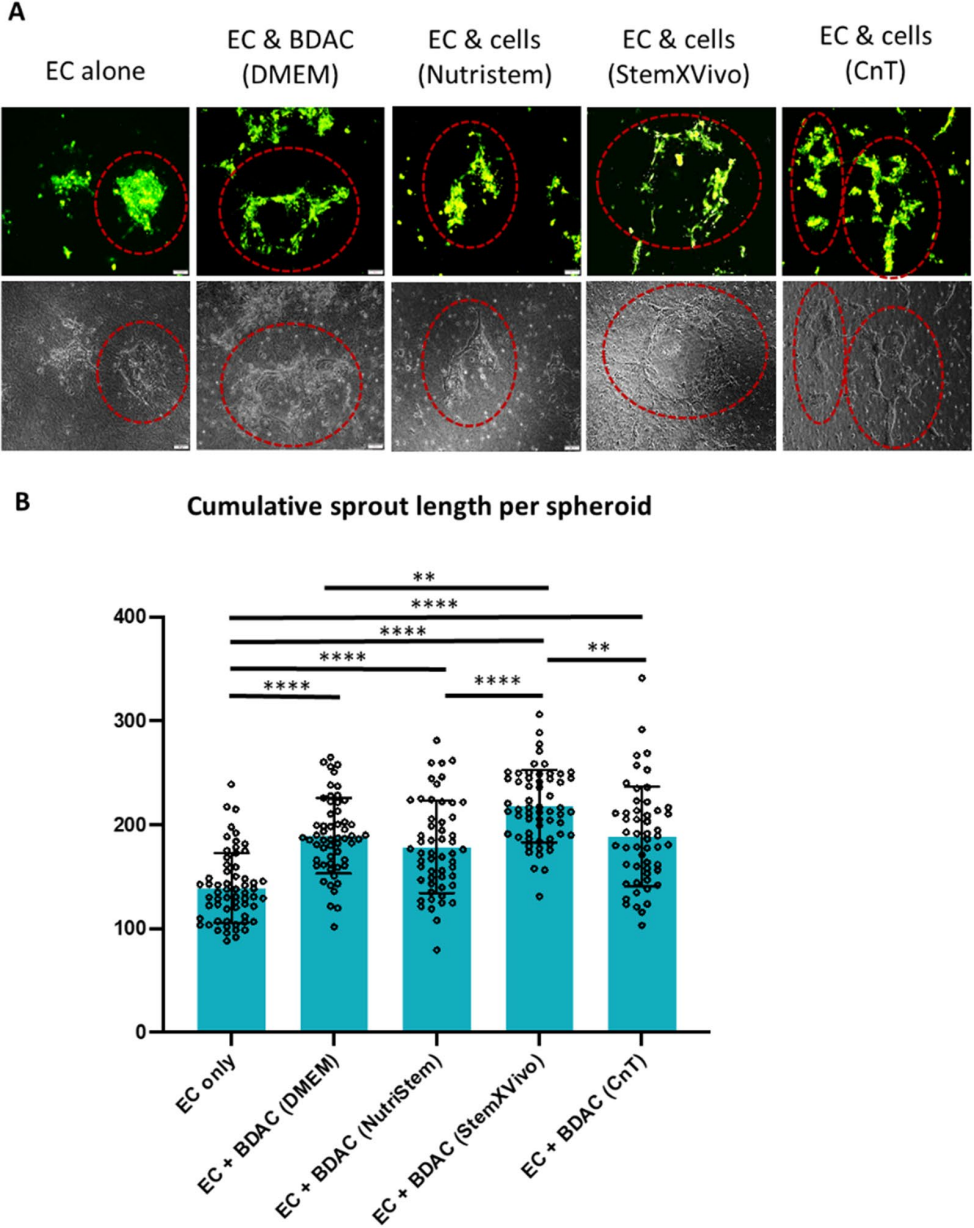
Adherent cells sourced from PBMCs under XF/SF conditions enhanced endothelial sprouting. GFP-expressing endothelial cell spheroids were embedded within collagen I hydrogels with optional supplementation of cells sourced from PBMCs in various media and allowed to sprout for 1 day. **A** Phase contrast and fluorescent micrographs were taken at various locations and representative sprouting spheroids are highlighted in red circles. **B** Quantification of cumulative sprout length per spheroid cultured alone or in co-culture with PBMC-derived cells sourced in various media (*n* = 48)

The functionality of the sourced cells was investigated in a functional in vitro angiogenesis assay (Fig. 2). For this, endothelial cell spheroids were co-seeded with PBMC-derived cells at a concentration of 2 × 10^5^ cells/ml in 3D collagen I hydrogels and exposed to pro-angiogenic stimuli. The endothelial cells formed sprouts, which extend into the surrounding microenvironment (Fig. 2A), and their cumulative sprout length per spheroid was quantified (Fig. 2B). This served as a measure of the pro-angiogenic potential of the co-seeded cells [9, 24, 36, 37]. Supplementation of PBMC-derived cells to this functional assay resulted in an overall enhancement of endothelial sprouting, irrespective of the media the cells were sourced in. Moreover, the angiogenic potential of cells cultured in DMEM/FBS was comparable to that of cells sourced in NutriStem and CnT and was slightly exceeded by that of cells sourced in StemXVivo (Fig. 2). As sourcing of adherent cells in StemXVivo resulted in lower cell numbers, while cells sourced in NutriStem exhibited a decreased expression of CD11b, cells sourced in CnT exhibited the closest similarity to cells sourced in DMEM/FBS. Hence, adherent cells sourced in CnT were considered to resemble BDACs and were used in all further experiments.

### BDACs exhibit superior ability to promote revascularization

BDACs sourced under xeno-free conditions (in CnT medium) were investigated for their therapeutic potential to enhance reperfusion in a pre-clinical CLI model. Human bone marrow-derived MSCs cultured under standard culture conditions were used as a state-of-the-art control.

**Fig. 3:**
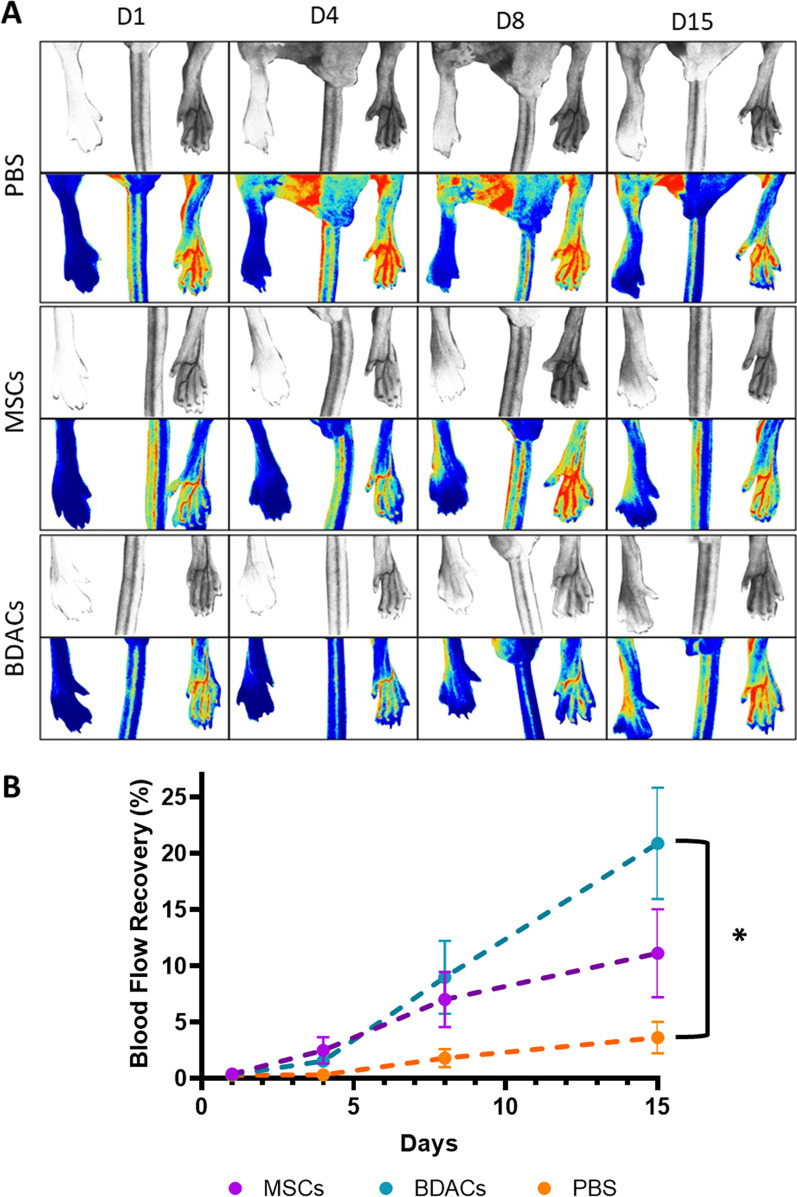
Mapping blood perfusion In ischemic limbs after ligation of the external iliac artery and intramuscular injection of PBS, MSCs or BDACs (*n* = 6). **A** Representative micrographs of laser speckle measurements of the real-time blood reperfusion of affected and healthy limbs across the three treatment groups. **B** Quantification of blood flow recovery of the affected hindlimb, displayed as percentages of limb perfusion of the non-operated limb

**Fig. 4:**
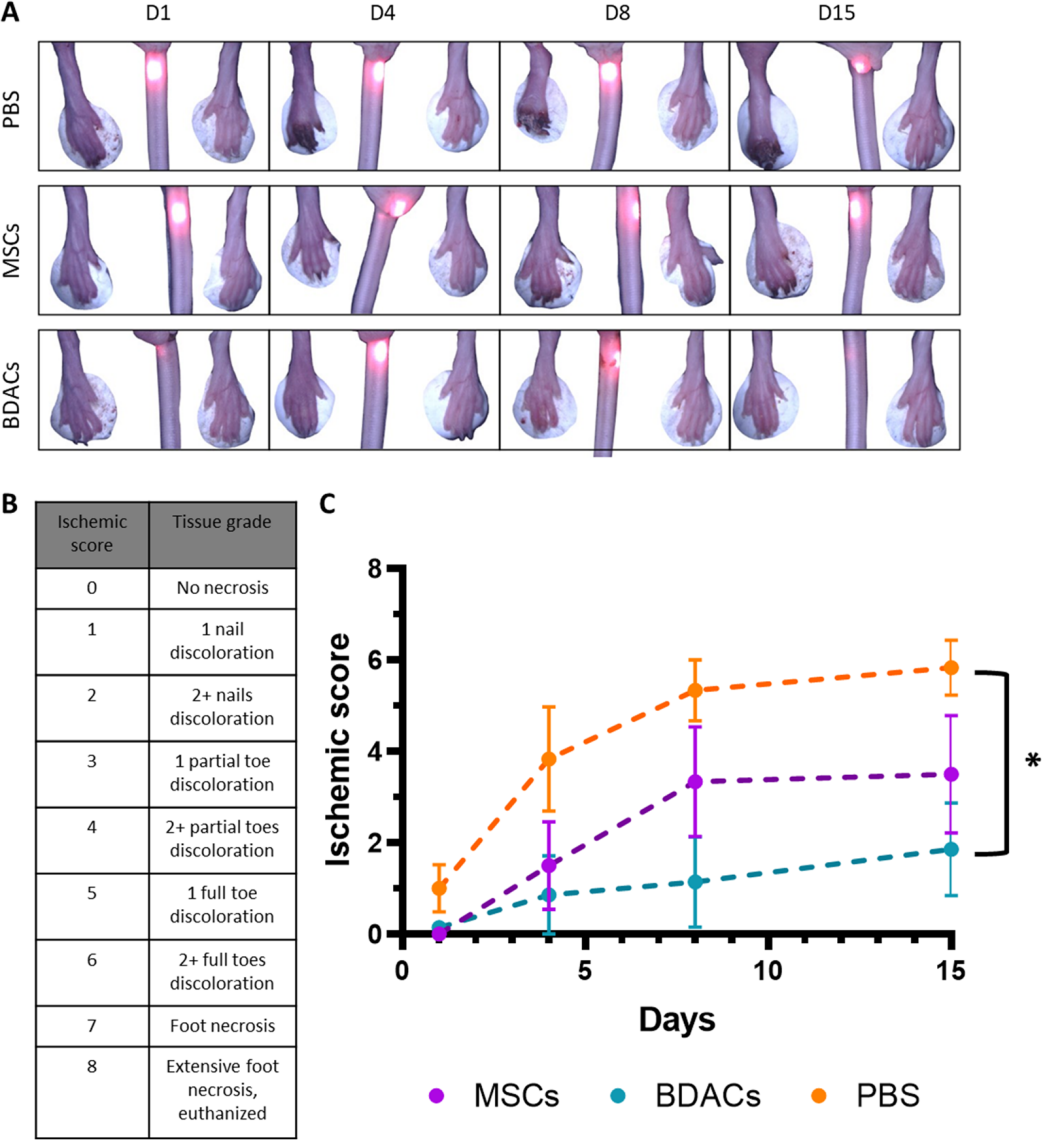
Semi-quantitative analysis of the ischemic score of murine hindlimbs after ligation of the external iliac artery and intramuscular injection of PBS, MSCs or BDACs (*n* = 6). **A** Representative micrographs of the ischemic and healthy limb (control) for each treatment group. **B** Classification of ischemic score. **C** Plotted ischemic score over time

Immunodeficient nude mice underwent external iliac artery ligation on the right limb, while the left served as the unaffected control. BDACs, MSCs or the empty vehicle (PBS) were injected intramuscularly around the ligation site immediately after ligation and before skin closure. Complete ligation was confirmed on day 1 and reperfusion was monitored on day 4, 8, and 15 post-surgery by laser speckle imaging (Fig. 3A). Obtained images were used to quantify the percentage of reperfusion as compared to the control limb (Fig. 3B). Moreover, representative photographs of the limbs were also recorded at each time point to monitor any development/progression of necrosis (Fig. 4A). These were used to semi-quantify the ischemic score of animals undergoing various treatments (Fig. 4 B, C).

Among all treatment groups, animals that received BDACs intramuscularly showed an approximately fivefold increase in blood flow recovery over the first 2 weeks, as compared to vehicle control. Indeed, BDACs treatment resulted in the highest blood flow recovery rate of approximately 20%, followed by MSCs treatment (approximately 10%) and PBS vehicle (approximately 4%) (Fig. 3B).

The PBS-injected mice displayed a severe range of necrosis with an average ischemic score of 6, indicating 2 or more full toes with discoloration (Fig. 4A–C). Mice treated with MSCs showed a more moderate necrotic profile but had a largely inconsistent range of necrosis across the groups. An average ischemic score of 3 was seen, but some cases had discoloration of 1 or more toes while others had only 2 or more nail discolorations at most. For the BDACs-treated group, a comparatively consistent ischemic profile was observed, with most mice only showing very minor signs of necrosis. The worst outcome in any of the BDACs-treated mice was an ischemic score of 2, indicating 2 or more nails being discolored (Fig. 4A–C).

**Fig. 5:**
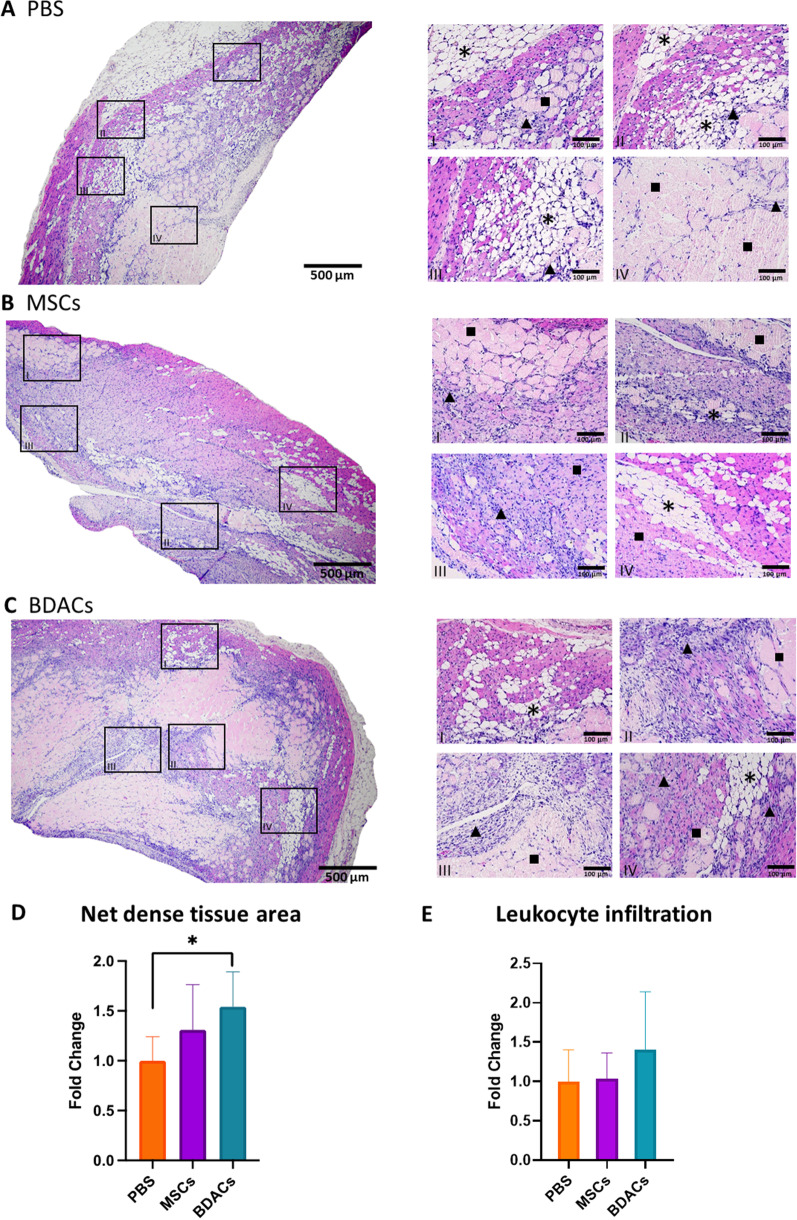
H&E staining of the murine calf gastrocnemius muscle of the following groups **A** PBS, **B** MSCs, and **C** BDACs (*n* = 6). Enlarged sections of the representative images show mononucleated cell infiltration (denoted by triangles), adipose replacement (denoted by stars), and muscle degeneration (denoted by squares). **D** Quantification of average dense tissue area, expressed as fold-change to PBS samples. **E** Leukocyte infiltration of tissue was measured as fold-change of the neutrophil cell count to average tissue area between PBS and other two conditions

**Fig. 6:**
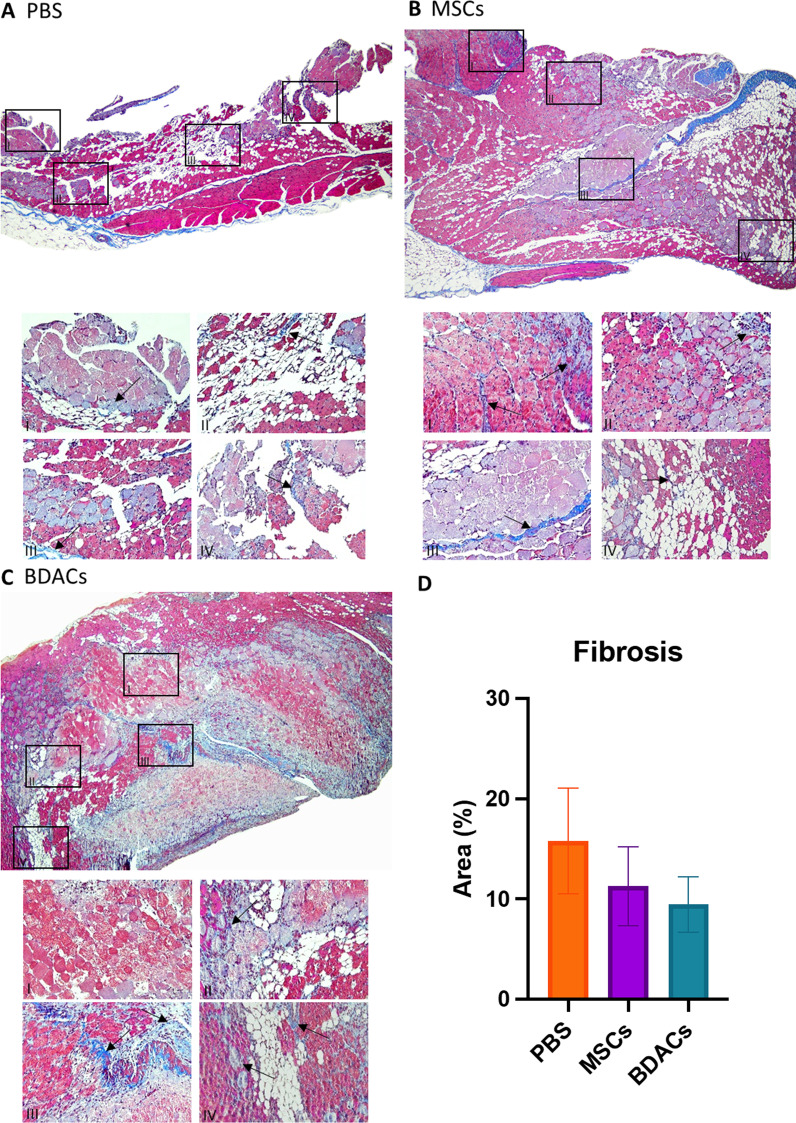
Masson’s trichrome staining of murine gastrocnemius muscle in **A** PBS, **B** MSCs, and **C** BDACs-treated groups (*n* = 6). Enlarged sections of the representative images show fibrosis (indicated via arrows) and muscle degeneration

On day 15, mouse gastrocnemius muscle tissues from each treatment group were harvested, fixed and paraffin-embedded for histological analysis. Hematoxylin and Eosin (H&E) staining allowed to visualize and semi-quantify the tissue morphology, such as cell infiltration around muscle fibers (indicator of inflammation) and adipose replacement (suggestive of muscle tissue degeneration) (Fig. 5). Masson’s trichrome staining allowed for the differentiated visualization of fibrosis by staining the collagen fibers in blue, as well as semi-quantification of fibrosis (Fig. 6).

Animals in all three treatment groups predictably exhibited certain morphologies of ischemic tissue such as increased inflammation and muscle degeneration (Fig. 5). H&E-stained muscle tissue from the vehicle treatment group showed an overall higher degree of adipose replacement and degeneration, as indicated by the smallest dense tissue (all tissue except adipose) area (Fig. 5D), while it exhibited similar levels of cell infiltration as the MSC treatment group (Fig. 5E). Moderate amounts of adipose replacement were observed in muscle tissue derived from animals of the MSCs treatment group (Fig. 5D, E). In accordance with the ischemic score and limb perfusion rates, muscles from the BDAC treatment group showed the least degrees of adipose replacement, while the levels of inflammatory cell infiltration were elevated, albeit not significantly (Fig. 5D, E).

Masson’s trichrome staining of muscle tissue isolated from animals of all groups showed various degrees of tissue fibrosis, either diffusively present around muscle fibers or taking up distinct elongated tissue sections (Fig. 6A–C). Although not statistically significant, the largest area percentage covered by blue-stained fibrosis was found in the PBS vehicle group, whereas lower levels of fibrosis were observed in the MSCs treatment group. The BDACs treatment group exhibited the lowest levels of fibrosis (Fig. 6D).

**Fig. 7:**
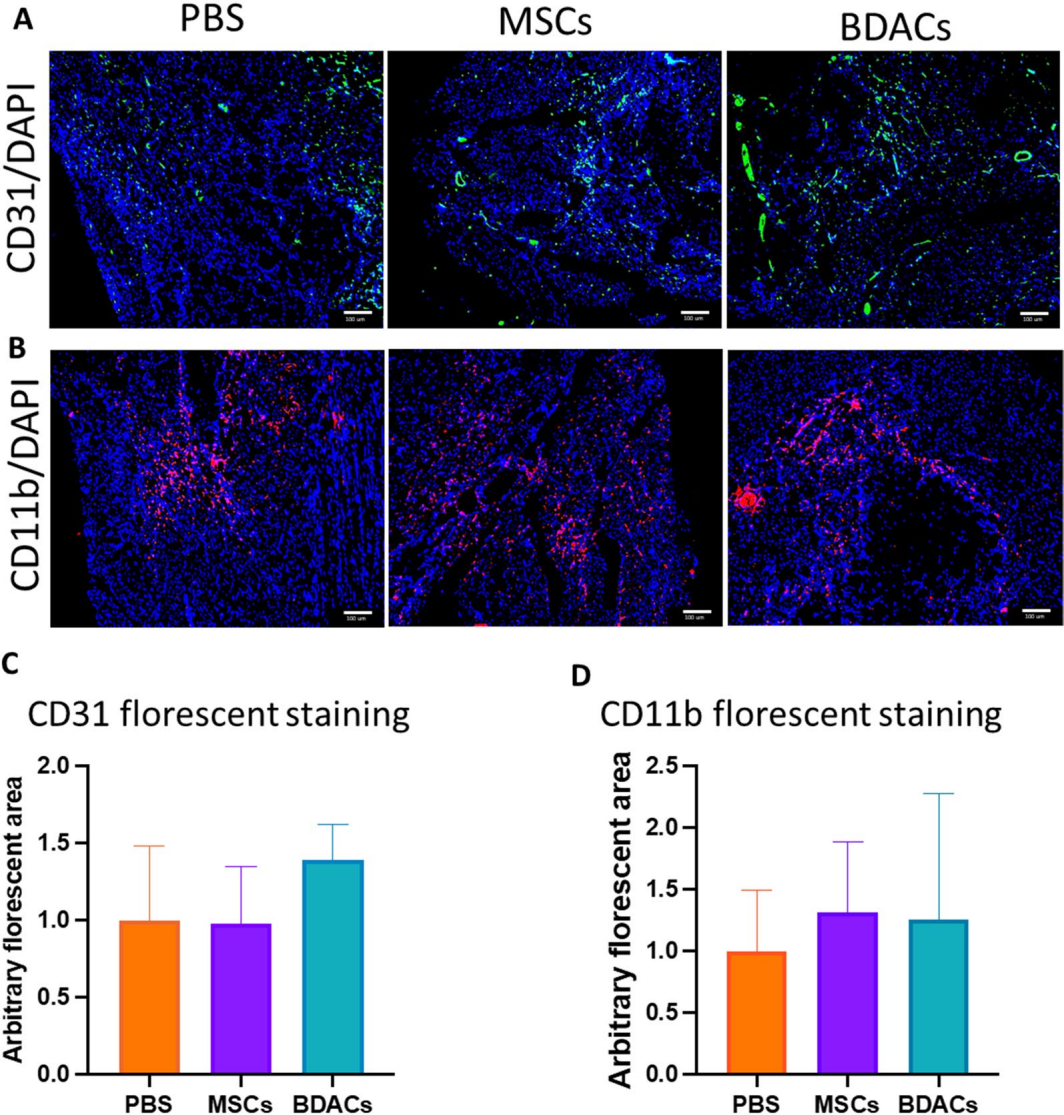
Representative images of immunohistochemically stained murine gastrocnemius muscle for CD31 (green) and CD11b (red) (*n* = 6). **A** DAPI was used to visualize nuclei staining (blue). **B** Quantification of CD11b- and **C** CD31-stained area of histological sections of muscle tissue derived from mice in the MSCs and BDACs treatment and vehicle groups

Tissue sections were next subjected to immunohistochemical evaluation of vessel density by staining for CD31 (Fig. 7A) and monocyte infiltration by staining for CD11b (Fig. 7B). The stained area was quantified and normalized to the DAPI area of the respective imaged areas and plotted as fold- change as compared to the vehicle group (Fig. 7C, D). No obvious differences were observed in the degree of infiltrated myeloid cells (Fig. 7C), while a trend toward higher microvessel density was seen in muscle tissue from the BDACs treatment group. Due to the high variability between samples, these differences were not significant.

**Fig. 8:**
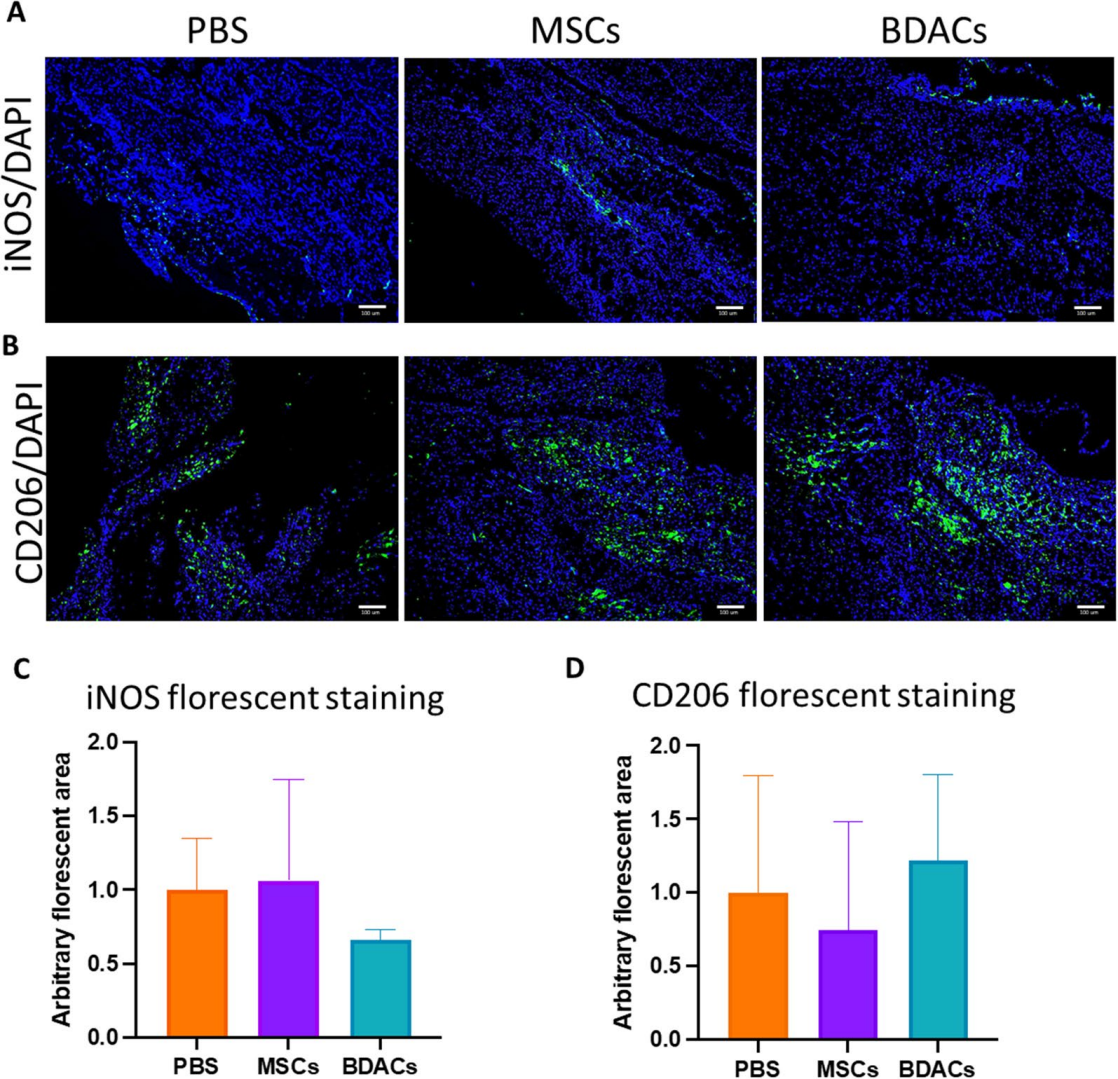
Representative images of immunohistochemically stained murine gastrocnemius muscle tissues for iNOS (green) and CD206 (green) (*n* = 6). **A** DAPI was used to visualize cell nuclei (blue). **B** Quantification of iNOS- and **C** CD206-stained area of histological sections of muscle tissue derived from mice in the MSCs and BDACs treatment and vehicle groups

Similarly, tissue sections were also immunohistochemically investigated for the presence of pro-inflammatory (iNOS; Fig. 8A, C) and anti-inflammatory (CD206; Fig. 8B, D) macrophages. Again, no significant differences were found, although a trend toward lower levels of pro-inflammatory macrophages was observed in tissues of the BDACs treatment group, while CD206 levels varied strongly in between samples (Fig. 8A–D).

### BDAC secretome is enriched in pro-angiogenic factors

**Fig. 9:**
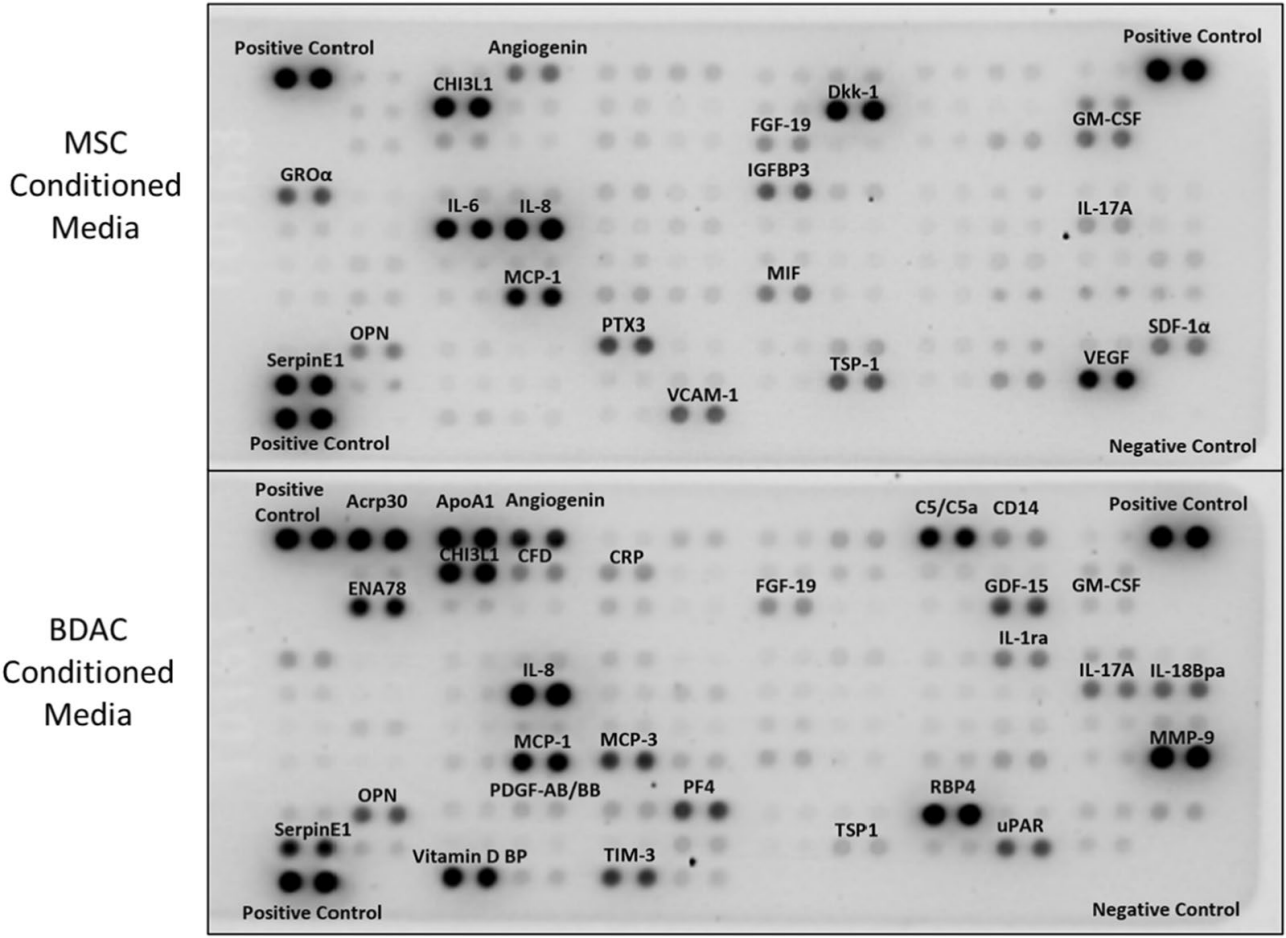
Proteome arrays of proteins secreted by MSCs and BDACs. Strong signals are labeled on the membrane

Both treatments based on MSCs and BDACs demonstrated the potential to promote revascularization and impair the development and/or progression of tissue degeneration and foot necrosis. Nonetheless, BDACs’ pro-angiogenic potential exceeded that of MSCs, suggesting differences in quality or quantity of secreted factors. In order to compare the different secretomes of MSCs and BDACs, media conditioned by each cell type, respectively, were analyzed for secreted human proteins using a commercially available proteome profiler membrane. A variety of factors was identified in both cellular secretomes revealing that the overall secretion profile differed (Fig. 9).

**Fig. 10:**
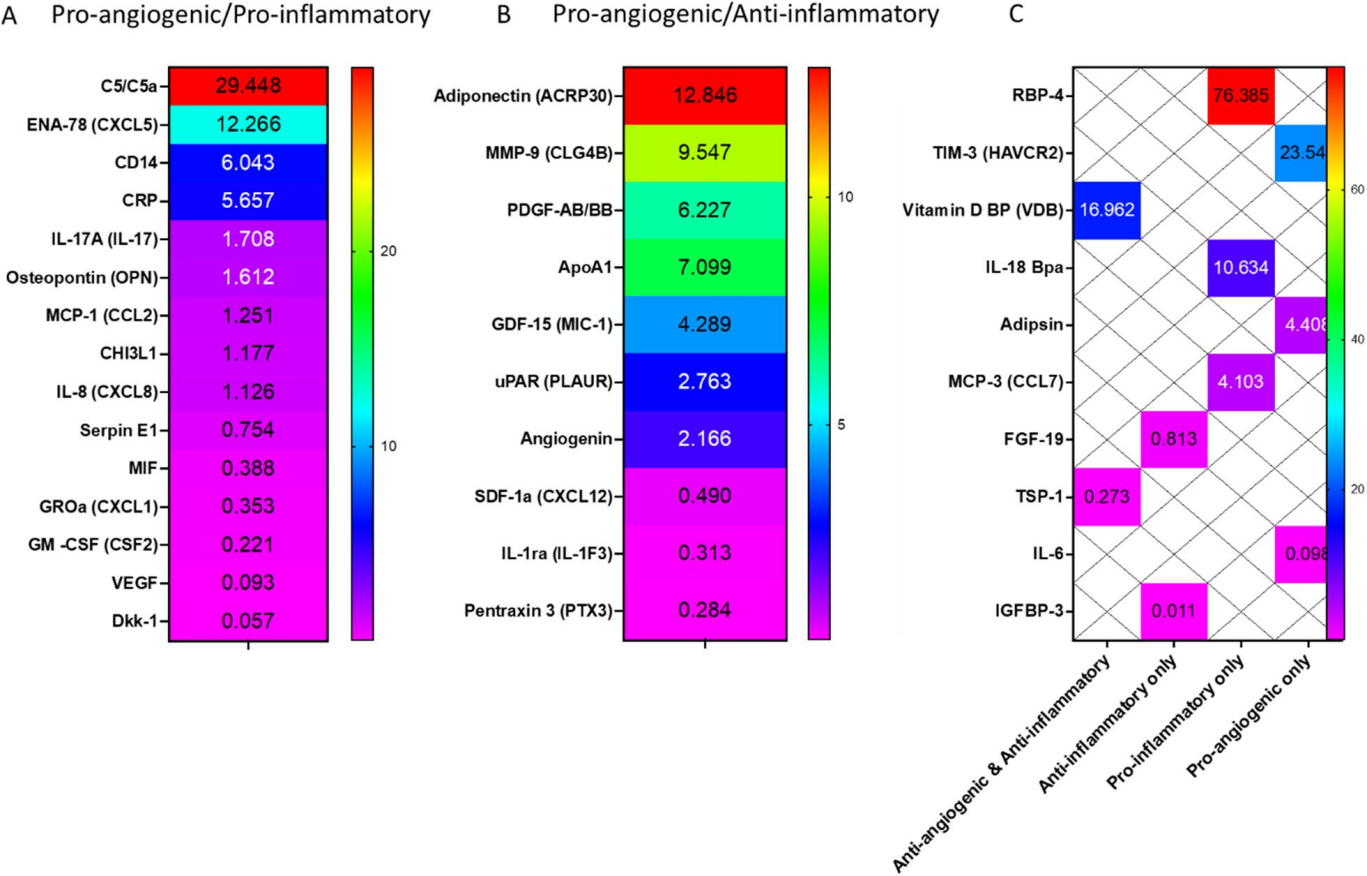
List of secreted proteins secreted by BDACs and/or MSCs. Signals on proteome array membranes were quantified densitometrical and plotted as a heatmap of fold changes of signal intensities BDACs/MSCs. Cytokines identified by the proteome profiler array are categorized into: **A** pro-angiogenic and pro-inflammatory, **B** pro-angiogenic and anti-inflammatory, **C** other combinations of bioactivities

BDAC secreted highest amounts of pro-angiogenic and pro-inflammatory factors such as CXCL5 (ENA78), IL17 and osteopontin (OPN), while factors with similar bioactivity attributes including MIF, CXCL1 (GROa), GM-CSF and VEGF were found enriched in the MSC secretome. BDACs and MSCs shared also similar levels of MCP-1 and IL8 (Fig. 10A). A larger number of factors with pro-angiogenic, but anti-inflammatory properties were enriched in the BDAC secretome including adiponectin, MMP-9, PDGF-AB/BB, MIC-1, uPAR and angiogenin, while CXCL12 and pentraxin 5 (TSG-14) were more abundant in the MSC-derived secretome (Fig. 10B). Interestingly, proteases and their respective inhibitors such as MMP-9, uPAR and TIM were also most enriched in BDAC conditioned media (Fig. 10B, C), suggesting that BDAC may play an active role in tissue remodeling. Only one anti-angiogenic and anti-inflammatory factor has been identified in BDAC- and MSC-derived secretomes, respectively. Here, BDACs secreted alleviated levels of VDB, while MSCs exhibited increased secretion levels of TSP-1. In addition, the MSC secretome was enriched in anti-inflammatory FGF-19 and IGFBP3, as well as in pro-angiogenic IL6, while BDACs secreted higher levels of pro-angiogenic adipsin and pro-inflammatory factors such as RBP4, IL18-BP and CCL7.

## Discussion and conclusion

BDACs isolated under xeno-free conditioned were able to significantly enhance reperfusion and reduce necrosis in a murine model of CLI, exceeding the therapeutic potential of MSCs.

Except for the chemically defined medium and the xeno- and serum-free medium, all media produced pro-angiogenic cells, indicating that the presence of some serum components may be indeed necessary for their sourcing. Although cells with the most prominent spindle-shaped cell morphology exhibited the strongest pro-angiogenic potential, slight deviations in cell morphology still resulted in pro-angiogenic cells. Similarly, decreased expression of CD11b did not affect pro-angiogenic potential of the sourced cells. Nonetheless, culture medium enabling the sourcing of cells, which most closely resembled BDACs sourced under standard culture conditions in terms of cell morphology, marker expression, as well as maximal number of cells being sourced, was chosen for further studies. Hence, a protocol to source BDACs under xeno-free conditions was established.

When the therapeutic potential of BDACs was compared to that of MSCs, which is the most commonly investigated cell type for the treatment of CLI [7, 42], BDACs enhanced reperfusion of the affected limb twofold more as compared to MSCs. This difference was not statistically significant and further experiments may be needed to compare the pro-angiogenic potential of BDACs to that of MSCs. Nonetheless, this is likely the reason behind the significant reduction in necrosis and tissue degeneration in BDAC-treated animals and BDACs’ superior potential to salvage the affected limb. This was also obvious on tissue level, where treatment with BDACs significantly reduced muscle tissue shrinkage. Since levels of fibrosis were highest in the PBS-treated group and slightly elevated in the MSC-treated group as compared to the BDAC-treated group, BDACs may attenuate fibrotic responses. This may either happen directly, as BDACs secreted various anti-inflammatory factors, or indirectly by promoting angiogenesis and thereby salvaging tissue from more damage, which would otherwise lead to increased fibrosis. Both effects were observed by histology, as trends toward higher levels of CD31-stained microvessels and a lower ratio of M1-to-M2 macrophages may indicate. Due to high animal-to-animal variations, differences in these parameters did not exhibit statistical significance. Furthermore, larger differences in microvessel density would be expected at an earlier time point during the early proliferation phase, as vessels are known to regress during tissue remodeling toward fibrosis [43]. The immune-compromised status of the animals that will result in overall attenuated levels of immune cells [44], is also a likely explanation for the lack of statistical significances in the levels of various macrophage populations. Nonetheless, concurring trends were observed, which suggest that healing processes are facilitated by BDACs, resulting in the rescue of the affected tissue.

The apparently stronger pro-angiogenic and pro-healing potential of BDACs as compared to the current gold standard, bone marrow-derived MSCs is not entirely surprising, as previously performed functional in vitro assays of endothelial sprouting have suggested as much [23].

As it is currently believed that cells mediate their effects via paracrine factors [45], we compared the secretomes of BDACs and MSCs. Indeed, the secretomes differed significantly, which is likely explained by their different origins [23]. It is noteworthy that overall the BDAC secretome contained a larger number of enriched factors with pro-angiogenic and anti-inflammatory properties. Simultaneously, the number of anti-angiogenic and pro-inflammatory factors was leveled out in both secretomes. Current data thus suggest that the enhanced therapeutic effects seen with BDACs are a result of the synergy of the combination of all those enriched factors. It remains to be shown, which of those factors are most relevant for the observed effect.

Mesenchymal stem cells (MSCs) were first isolated in 1976 from bone marrow [46]. Since then, they were well characterized and widely explored for the treatment of various pathologies. This does not necessarily mean that they are the most effective cell type for the treatment of a specific disease. With an outlook on angiogenesis mesenchymal pericytes, which are in essence MSCs, detach from vessels upon initiation of angiogenesis and are mainly involved in later stages, such as vessel maturation, proper function and quiescence [47]. Indeed, impairment of pericyte recruitment had no effect on microvascular density during embryonic development [48]. Hence, cells that are actively involved in the early stages of angiogenesis, such as pericytic cells of hematopoietic origin, may be more potent in promoting revascularization. BDACs closely resemble these cells that have been described in vivo so far [20, 49]. Cells of the myeloid lineage have been described to be major drivers of arteriogenesis [50] and anastomosis [51], two processes crucial during tissue revascularization and also relevant for tissue recovery in CLI. Furthermore, various types of MACs have shown therapeutic effects in CLI, while sharing certain characteristics with BDACs such as cellular origin and some makers [52, 53]. Since BDACs are of myeloid origin [24], they may also participate in these processes, but this remains to be shown. Nonetheless, the combination of both cell types for the treatment of CLI may have synergistic effects and this should be explored in future studies.

Autologous cell-based therapy is highly advantageous, since it minimized the risks of immunological rejection and disease transmission and as such faces less regulatory hurdles during translation into a clinical setting. Nonetheless, it also faces various difficulties and limitations at this point of time, including risk and discomfort of the patient during cell harvest, limited number of harvested cells that necessitate long expansion times, often resulting in decreased cell functionality and the difficulty to meet the therapeutic time window.

Here, we have shown that BDACs can be derived from an easy-accessible cell source, namely human peripheral blood, in clinically relevant numbers and time frame. The ease of sourcing would allow to harvest and utilize the patient’s own cells repeatedly. The successful sourcing of BDACs under xeno-free conditions will further pave the way for their clinical application.

## Data Availability

The datasets used and/or analyzed during the current study are available from the corresponding author on reasonable request.

## Abbreviations

BDACs: Blood-derived angiogenic cells
BSA: Bovine serum albumin
CLI: Critical limb ischemia
DMEM: Dulbecco’s modified Eagle medium
EC: Endothelial cell
ECFC: Endothelial colony-forming cell
ELC: Endothelial-like cell
EPC: Endothelial progenitor cell
FBS: Fetal bovine serum
GMP: Good Manufacturing Practice
HUVECs: Human umbilical vein endothelial cells
H&E: Hematoxylin–eosin
MAC: Myeloid angiogenic cell
MSC: Mesenchymal stem cell
M&T: Masson’s trichrome
OEC: Outgrowth endothelial cell
PAD: Peripheral artery disease
PBMCs: Peripheral blood mononucleated cells
P/S: Penicillin–Streptomycin
ROI: Region of interest
SF: Serum free
XF: Xeno free
